# Quantitative Computed Tomography (QCT) derived Bone Mineral Density (BMD) in finite element studies: a review of the literature

**DOI:** 10.1186/s40634-016-0072-2

**Published:** 2016-12-09

**Authors:** Nikolas K. Knowles, Jacob M. Reeves, Louis M. Ferreira

**Affiliations:** 1 Graduate Program in Biomedical Engineering, The University of Western Ontario, 1151 Richmond St, London, ON Canada; 2 Roth|McFarlane Hand and Upper Limb Centre, Surgical Mechatronics Laboratory, St. Josephs Health Care, 268 Grosvenor St, London, ON Canada; 3 Collaborative Training Program in Musculoskeletal Health Research, and Bone and Joint Institute, The University of Western Ontario, 1151 Richmond St, London, ON Canada; 4 Department of Mechanical and Materials Engineering, The University of Western Ontario, 1151 Richmond St, London, ON Canada

**Keywords:** QCT, Bone density, Finite element analysis, Mechanical properties

## Abstract

**Background:**

Finite element modeling of human bone provides a powerful tool to evaluate a wide variety of outcomes in a highly repeatable and parametric manner. These models are most often derived from computed tomography data, with mechanical properties related to bone mineral density (BMD) from the x-ray energy attenuation provided from this data. To increase accuracy, many researchers report the use of quantitative computed tomography (QCT), in which a calibration phantom is used during image acquisition to improve the estimation of BMD. Since model accuracy is dependent on the methods used in the calculation of BMD and density-mechanical property relationships, it is important to use relationships developed for the same anatomical location and using the same scanner settings, as these may impact model accuracy. The purpose of this literature review is to report the relationships used in the conversion of QCT equivalent density measures to ash, apparent, and/or tissue densities in recent finite element (FE) studies used in common density-modulus relationships. For studies reporting experimental validation, the validation metrics and results are presented.

**Results:**

Of the studies reviewed, 29% reported the use of a dipotassium phosphate (K_2_HPO_4_) phantom, 47% a hydroxyapatite (HA) phantom, 13% did not report phantom type, 7% reported use of both K_2_HPO_4_ and HA phantoms, and 4% alternate phantom types. Scanner type and/or settings were omitted or partially reported in 31% of studies. The majority of studies used densitometric and/or density-modulus relationships derived from different anatomical locations scanned in different scanners with different scanner settings. The methods used to derive various densitometric relationships are reported and recommendations are provided toward the standardization of reporting metrics.

**Conclusions:**

This review assessed the current state of QCT-based FE modeling with use of clinical scanners. It was found that previously developed densitometric relationships vary by anatomical location, scanner type and settings. Reporting of all parameters used when referring to previously developed relationships, or in the development of new relationships, may increase the accuracy and repeatability of future FE models.

## Background

Accurate characterization of the properties of bone in finite element (FE) studies, including accurate local bone density (Schileo et al. 2008; Synek et al. 2015), is essential to improve the accuracy of existing continuum-level FE modeling techniques (Schileo et al. 2008). Uncalibrated clinical CT images are limited to voxel information in the form of x-ray absorption coefficients, using the Hounsfield (HU) scale, with air (−1000 HU) and water (0 HU) as references. For high atomic number materials, quantitative computed tomography (QCT) provides local densitometric measurements in volumetric bone mineral density (vBMD) (Engelke et al. 2013). This allows for accurate regional variations in BMD to be mapped in subsequent continuum-level finite element models (FEMs). The accuracy and characterization of using calibration phantoms has been well established over the past two decades (Faulkner et al. 1993; Keyak et al. 1994; Les et al. 1994; Schileo et al. 2008).

**Fig. 1:**
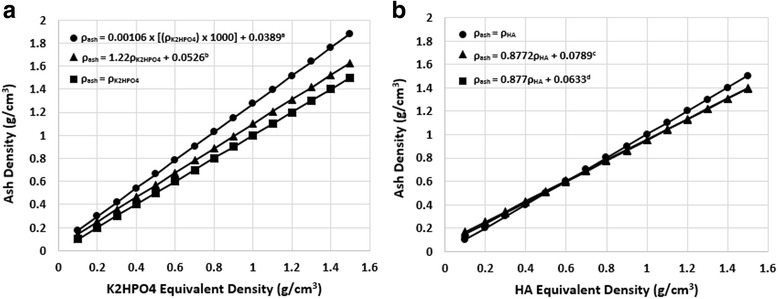
Ash and QCT equivalent density (**a:** dipotassium phosphate; **b:** calcium hydroxyapatite) relationships used in reviewed studies. Relationships from: ^a^ (Keyak et al. 1994) – 140 kVp, 70 mA; ^b^ (Les et al. 1994) – 140 kVp, 30 mA; ^c^ Unknown – used in (Eberle et al. 2013a, b); ^d^ (Keyak et al. 2005) – 80 kVp, 280 mAs

Calibrated vBMD or quantitative equivalent CT density (ρ_QCT_) is calculated by measuring the CT scanner’s response to the phantom’s calibrated regions. Typical calibration phantoms contain rods with varying concentrations of calcium hydroxyapatite (HA) (Engelke et al. 2013; Poelert et al. 2013), or are calibrated using liquid dipotassium phosphate (K_2_HPO_4_), and provide equivalent density in units of mg_HA_/cm^3^ (ρ_HA_) or ${\mathrm{mg}}_{{\mathrm{K}}_2\mathrm{H}\mathrm{P}{\mathrm{O}}_4}/{\mathrm{cm}}^3\left({\uprho}_{{\mathrm{K}}_2\mathrm{H}\mathrm{P}{\mathrm{O}}_4}\right)$(Keyak et al. 1994; Les et al. 1994). These imaging based density methods have been related to physical methods, such as ash density (ash mass divided by bulk sample volume), and apparent density (wet mass divided by bulk sample volume) by use of CT scan energy specific (linear) relationships (Fig. 1) (Faulkner et al. 1993; Giambini et al. 2015).

To account for the lack of cancellous bone geometry due to the clinical CT resolution, continuum-level FEMs use spatial variations of BMD related to mechanical properties in order to achieve physiologic accuracy. In the development of these FEMs, two relationships are required to convert raw CT x-ray attenuation data to bone mechanical properties. The first densitometric relationship relates raw CT attenuation to BMD (ρ = a*HU + b) (ρ_QCT_ if phantom calibrated), and the second mechanical property relationship, relates BMD to bone mechanical properties. To develop the second relationship, most studies use relationships developed using physical specimens and have found continuous functions and power relationships best fit experimental data (E = αρ^β^), where E is the Young’s Modulus, α and β are experimentally derived parameters, and ρ is the bone density (Helgason et al. 2008). Alternatively, relationships may be piecewise functions that represent experimentally derived relationships for cancellous and cortical bone separately. Density-modulus relationships for cancellous and cortical bone are determined by the experimental method in which they are derived. Small bone sample are typically mechanically tested to derive the desired relationships. Many of these studies test cancellous samples and cortical samples separately (instead of whole bones), and therefore derive separate equations for each bone type (Rice et al. 1988; Schaffler and Burr 1988). Due to the experimental testing of physical specimens, these equations use physical BMD measures such as ash, apparent, or tissue density; and therefore when using QCT derived equivalent density (ρ_QCT_), conversions between QCT, ash (ρ_ash_), apparent (ρ_app_), and tissue densities (ρ_tissue_) are required for accurate FEM development.

Experimentally derived density-modulus relationships are site-specific (Morgan et al. 2003; Schileo et al. 2008), and are also affected by the quality and pathology of the bone, with density being a function of the CT scanner settings (Faulkner et al. 1993). Therefore, the purpose of this literature review is to report i) the relationships used in the conversion of QCT equivalent density (ρ_QCT_) measures to ash (ρ_ash_), apparent (ρ_app_), and/or tissue densities (ρ_tissue_) in recent FE studies, and ii) the combined densitometric and density-modulus relationships impact on FEM accuracy.

## Methods

The specific relationships used in the conversion of QCT (K_2_HPO_4_ or HA) to physical density (ash, apparent, or tissue) in current FE studies were reviewed. The search was limited to FE studies of human bone published after January 1st, 2010, reporting clinical scanner image acquisition with use of a calibration phantom. Studies reporting only HR-pQCT or micro-CT scanner image acquisition were omitted. Literature searches included the search terms “finite element analysis, FE, or finite element” with combinations of “quantitative computed tomography,” “QCT,” and “bone.” Included articles represented a variety of calibration phantom types, anatomical locations, CT scanner settings, and density relationships and density-modulus relationships. Each article was carefully reviewed by one of two independent reviewers (NKK & JMR), and characterized based on anatomical location, density calibration type and manufacturer, scanner, and scanner settings. Articles not reporting any of the above were included as long as they clearly defined use of a calibration phantom with a clinical scanner. All articles were secondly reviewed by a single author (NKK) for completeness, and to extract specific densitometric and density-modulus relationships reported in each study. At this stage, references reported for densitometric and density-modulus relationships were checked and collected. Discrepancies between reported relationships and accurate relationships were noted, and corrected, if possible. Validation metrics and results are included for studies comparing experimental to FEM results.

**Fig. 2:**
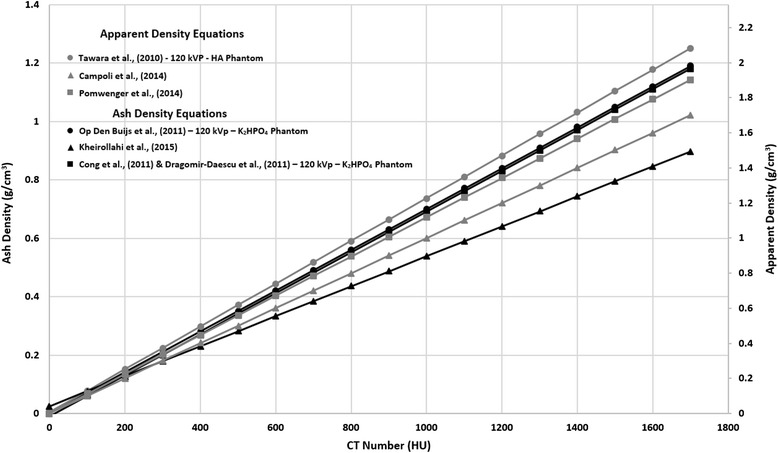
Apparent and ash density to CT number relationships reported by reviewed studies. Peak tube voltage and phantom type are reported when available. The relationship ρ_ash_ = 0.6ρ_app_ is assumed (Schileo et al. 2008)

The number of studies reporting each phantom type (Dipotassium Phosphate (K_2_HPO_4_), Hydroxyapatite (HA), both, other, or not reported), were determined along with manufacturer of the phantom. Of the studies reviewed, four relationships were noted (ash density from K_2_HPO_4_ density, ash density from HA density, ash density from CT number, or apparent density from CT number). Studies using these relationships were collected and plotted (Figs. 1 and 2). Density-modulus relationships were tabulated (Table 1), but not reviewed in detail, as this is beyond the scope of this review, and many are summarized in detail in the review by Helgason et al. (2008).

**Table 1:** Summary of Calibration Phantom, Densitometric and Modulus Relationships, Scanner and Scanner Settings

Author, Year	Anatomical Location	Phantom Type	Phantom Manufacturer	Densitometric Relationship (g/cm^3^)	Density-Modulus Relationship (MPa)	Validation Measure Experimental vs. FEM (Metric Value(s))	Scanner	Peak Voltage (kVp)	Tube Current (mA)/Time Product (mAs)	Voxel Dimensions (mm)
(Tarala et al. 2011)	Femur	HA	Image Analysis	ρ_HA_ = ρ_ash_	NR	Displacement	NR	NR	NR	NR
						CLS Stem R^2^ = 0.95 EPOCH Stem R^2^ = 0.88				
(Cong et al. 2011)	Femur	K_2_HPO_4_	Mindways	ρ_ash_ = ${\uprho}_{{\mathrm{K}}_2\mathrm{H}\mathrm{P}{\mathrm{O}}_4}$ = −0.009 + 0.0007 HU ρ_ash_/ρ_app_ = 0.6^a^		Axial Stiffness	Somatom Definition, Siemens	120	216 mAs	0.40 × 0.45 × 0.45
					E = 14664ρ_ash_^1.49^	R^2^(y = x) = −1.40				
					E = 10500ρ_ash_^2.29^	R^2^(y = x) = −4.97				
					E = 17546ρ_ash_^3^	R^2^(y = x) = −6.93				
					E = 8050ρ_ash_^1.16^	R^2^(y = x) = 0.50				
					E = 15000*e* ^-4.91*e*-2.63ρash^	R^2^(y = x) =0.71				
					E = 20000*e* ^*^* -5.19*e*-2.10ρash^	R^2^(y = x) = 0.69				
					E = 55000*e* ^*^* -5.40*e*-2.63ρash^	R^2^(y = x) = 0.69				
(Dragomir-Daescu et al. 2011)	Femur	K_2_HPO_4_	Mindways	ρ_ash_ = ${\uprho}_{{\mathrm{K}}_2\mathrm{H}\mathrm{P}{\mathrm{O}}_4}$ = −9*10^−3^ + 7* 10^−4^*HU ρ_ash_/ρ_app_ = 0.6^a^	E = 14664ρ_ash_^1.49^	Axial Stiffness	Somatom Definition, Siemens	120	216 mAs	0.40 × 0.30 to 0.45
						R^2^ = 0.87				
						Ultimate Load				
						R^2^ = 0.93				
(Keyak et al. 2011)	Femur	HA	Image Analysis	NR	NR	NR	NR	120	140 mAs	NR
(Trabelsi and Yosibash 2011)	Femur	K_2_HPO_4_	NR	ρ_ash_ = 1.22${\uprho}_{{\mathrm{K}}_2\mathrm{H}\mathrm{P}{\mathrm{O}}_4}$ + 0.0523^b^	E_cort_ = 10200ρ_ash_^2.01^ E_trab_ = 5307ρ_ash_ + 469	Strain	NR	NR	NR	NR
						R^2^ = 0.982 empirical R^2^ = 0.939 MM-based				
(Trabelsi et al. 2011)	Femur	K_2_HPO_4_	Mindways	ρ_ash_ = 1.22${\uprho}_{{\mathrm{K}}_2\mathrm{H}\mathrm{P}{\mathrm{O}}_4}$ + 0.0523^b^	E_cort_ = 10200ρ_ash_^2.01^ E_trab_ = 5307ρ_ash_ + 469	Displacement	Lightspeed VCT, GE Healthcare	120	90 mAs	1.0 × 0.488 to 0.547
						R^2^ = 0.871				
						Strain				
						R^2^ = 0.951				
						Axial Stiffness				
						R^2^ = 0.619				
(Amin et al. 2011)	Femur	European Spine Phantom	NA	NR	NR	NE	Lightspeed QX/i, GE Healthcare	NR	NR	2.5 × 0.74 × 0.74
(Op Den Buijs and Dragomir-Daescu 2011)	Femur	K_2_HPO_4_	Mindways	ρ_ash_ = ${\uprho}_{{\mathrm{K}}_2\mathrm{H}\mathrm{P}{\mathrm{O}}_4}$ = 7.0*10^−4^HU^c^	E = 29800ρ_ash_^1.56^	Axial Stiffness	Somatom Definition, Siemens	120	216 mA	0.40 × 0.29 to 0.41
						R^2^ = 0.76				
						Strength				
						R^2^ = 0.71				
(Koivumäki et al. 2012a)	Femur	HA	Osteo	ρ_ash_ = ρ_HA_	E = 10095ρ_ash_	Fracture Load	Sensation 16, Siemens	120	100 mAs	0.75 × 0.25 × 0.25
						R^2^ = 0.87				
(Shim et al. 2012)	Femur	NR	NR	NR	E = 6750.3ρ_ash_^2.01^	NE	NR	NR	NR	NR
(Gong et al. 2012)	Femur	HA	Image Analysis	ρ_HA_ to ρ_app_ and converted to ρ_ash_^d^ – Equation NR	E = 0.001 for ρ_ash_ = 0 E = 33900ρ_ash_^2.20^ for 0 < ρ_ash_ < 0.27 E = 5307ρ_ash_ + 469 for 0.27 < ρ_ash_ < 0.60 E = 10200ρ_ash_^2.01^ for ρ_ash_ > 0.60	NE	Lightspeed 16, GE Healthcare	80	280 mA	2.5 × 0.9375 × 0.9375
(Tomaszewski et al. 2012)	Femur	HA	NR	ρ_ash_ = 0.0633 + 0.887ρ_HA_^e^	NR but referenced	NE	NR	NR	NR	NR
(Keaveny et al. 2012)	Femur	K_2_HPO_4_	Mindways	NR	NR but referenced	NE	NR	80	280 mAs	3.0 × 0.78 to 0.94 × 0.78 to 0.94
(Koivumäki et al. 2012b)	Femur	HA	Osteo	NR	NR	Cortical Fracture Load	Sensation 16, Siemens	120	100 mAs	0.75 × 0.25 × 0.25
						R^2^ = 0.73				
(Ruess et al. 2012)	Femur	NR	NR	${\uprho}_{{\mathrm{K}}_2\mathrm{H}\mathrm{P}{\mathrm{O}}_4}$ = 10^−3^(0.793)HU ρ_ash_ = 1.22${\uprho}_{{\mathrm{K}}_2\mathrm{H}\mathrm{P}{\mathrm{O}}_4}$ + 0.0523^b^	E_cort_ = 10200ρ_ash_^2.01^ E_trab_ = 5307ρ_ash_ + 469	Strain	Brilliance 64, Phillips	120	250 mAs	1.25 × 0.195 × 0.195
						R^2^ = 0.918–0.981 See paper for specifics by method				
(Eberle et al. 2013a)	Femur	K_2_HPO_4_	Mindways	ρ_ash_ = 1.22${\uprho}_{{\mathrm{K}}_2\mathrm{H}\mathrm{P}{\mathrm{O}}_4}$ + 0.0523^b^ ρ_HA_ = 1.15${\uprho}_{{\mathrm{K}}_2\mathrm{H}\mathrm{P}{\mathrm{O}}_4}$ - 0.0073^f^ ρ_ash_ = 0.8772ρ_HA_ + 0.0789 ρ_app_ = 1.58 ρ_ash_ + 0.00011		Strain	Lightspeed VCT, GE Healthcare	120	90 mAs	1.0 × 0.547 × 0.547 OR 1.0 × 0.488 × 0.488
					E = 10200ρ_ash_^2.01^	Bland-Altman (mean) −9%				
					E = 6850ρ_app_^1.49^	Bland-Altman (mean) −10.6%				
					E = 15100${\uprho}_{{\mathrm{K}}_2\mathrm{H}\mathrm{P}{\mathrm{O}}_4}^{2.225}$	Bland-Altman (mean) −7.9%				
						Displacement				
					E = 10200ρ_ash_^2.01^	Bland-Altman (mean) −20.9%				
					E = 6850ρ_app_^1.49^	Bland-Altman (mean) −22.9%				
					E = 15100${\uprho}_{{\mathrm{K}}_2\mathrm{H}\mathrm{P}{\mathrm{O}}_4}^{2.225}$	Bland-Altman (mean) 1.6%				
						Axial Stiffness				
					E = 10200ρ_ash_^2.01^	Bland-Altman (mean) 15.8%				
					E = 6850ρ_app_^1.49^	Bland-Altman (mean) 22.6%				
					E = 15100${\uprho}_{{\mathrm{K}}_2\mathrm{H}\mathrm{P}{\mathrm{O}}_4}^{2.225}$	Bland-Altman (mean) −9.6%				
(Eberle et al. 2013b)	Femur	K_2_HPO_4_	Mindways	ρ_ash_ = 1.22${\uprho}_{{\mathrm{K}}_2\mathrm{H}\mathrm{P}{\mathrm{O}}_4}$ + 0.0523^b^ ρ_HA_ = 1.15${\uprho}_{{\mathrm{K}}_2\mathrm{H}\mathrm{P}{\mathrm{O}}_4}$ - 0.0073^f^ ρ_ash_ = 0.8772ρ_HA_ +0.0789 ρ_app_ = 1.58 ρ_ash_ + 0.00011		Strain	Lightspeed VCT, GE Healthcare	120	90 mAs	1.0 × 0.547 × 0.547 OR 1.0 × 0.488 × 0.488
					E = 12486${\uprho}_{{\mathrm{K}}_2\mathrm{H}\mathrm{P}{\mathrm{O}}_4}^{1.16}$	Relative Error (mean) 5%				
					E = 8346ρ_app_^1.50^	Relative Error (mean) −28%				
					E = 8050ρ_ash_^1.16^	Relative Error (mean) 18%				
					E = 25000e^^ -5.40e-2.10ρash^	Relative Error (mean) −16%				
					E = 6850ρ_app_^1.49^	Relative Error (mean) −12%				
						Displacement				
					E = 12486 ${\uprho}_{{\mathrm{K}}_2\mathrm{H}\mathrm{P}{\mathrm{O}}_4}^{1.16}$	Relative Error (mean) −10%				
					E = 8346ρ_app_^1.50^	Relative Error (mean) −40%				
					E = 8050ρ_ash_^1.16^	Relative Error (mean) 3%				
					E = 25000e^-5.40e-2.10ρash^	Relative Error (mean) −29%				
					E = 6850ρ_app_^1.49^	Relative Error (mean) −26%				
						Stiffness (N/mm)				
					E = 12486 ${\uprho}_{{\mathrm{K}}_2\mathrm{H}\mathrm{P}{\mathrm{O}}_4}^{1.16}$	Relative Error (mean) 6%				
					E = 8346ρ_app_^1.50^	Relative Error (mean) 56%				
					E = 8050ρ_ash_^1.16^	Relative Error (mean) −6%				
					E = 25000e^-5.40e-2.10ρash^	Relative Error (mean) 31%				
					E = 6850ρ_app_^1.49^	Relative Error (mean) 28%				
(Haider et al. 2013)	Femur	K_2_HPO_4_	Mindways	ρ_ash_ = 0.00106${\uprho}_{{\mathrm{K}}_2\mathrm{H}\mathrm{P}{\mathrm{O}}_4}$ + 0.0389^g^ ρ_ash_/ρ_app_ = 0.6^b^	E = 6850ρ_app_^1.49^	NE	NR	NR	NR	0.5 × 0.49 × 0.49
(Dall’Ara et al. 2012)	Femur	HA	QMR	BMD to BV/TV from μCT	Relation to BV/TV – Equation NR	Axial Stiffness	Brilliance 64, Phillips	120	100 mAs	1.0 × 0.33 × 0.33
						Stance: R^2^ = 0.449 Side: R^2^ = 0.869				
(Nishiyama et al. 2013)	Femur	HA	B-MAS200	ρ_ash_ = ρ_HA_	E = 10500ρ_ash_^2.29^	Axial Stiffness	Discovery CT750HD, GE Healthcare	120	60 mAs	0.625 × 0.439 × 0.439
						R^2^ = 0.89				
						Failure Load				
						R^2^ = 0.81				
(Kersh et al. 2013)	Femur	HA	NR	BV/TV = 9.3BMD + 3 from μCT^h^	NR	NE	Brilliance 64, Phillips	120	100 mA	0.60 × 0.36 × 0.36
(Keyak et al. 2013)	Femur	HA	Image Analysis	ρ_ash_ = 0.0633 + 0.887ρ_HA_^i^	E_trab_ = 14900ρ_ash_^1.86^	NE	Sensation 4, Siemens	120	140 mAs	NR
(Hambli and Allaoui 2013)	Femur	HA	Osteo	ρ_HA_ = 6.932*10^−4^HU - 5.68*10^−4^ ρ_ash_ = 1.22${\uprho}_{{\mathrm{K}}_2\mathrm{H}\mathrm{P}{\mathrm{O}}_4}$ + 0.0523^b^	E = 33900ρ_ash_^2.20^ for 0 < ρ_ash_ < 0.27 E = 5307ρ_ash_ + 469 for 0.27 < ρ_ash_ < 0.60 E = 10200ρ_ash_^2.01^ for ρ_ash_ > 0.60	Fracture Load	Somatom Plus 4, Siemens	120	160 mAs	0.70 × 0.25 × 0.25
						R^2^ = 0.943				
(Carballido-Gamio et al. 2013)	Femur	Both	Mindways & Image Analysis	NR	NR	NE	Sensation, Siemens	NR	NR	2.5 × 0.74 × 0.74 & 1.0 × 0.98 × 0.98
(Nishiyama et al. 2014)	Femur	Both	Mindways & B-MAS200	ρ_ash_ = ρ_HA_	E = 10500ρ_ash_^2.29^	NE	Somatom Cardiac 64, Siemens	120	250 mAs	0.50 × 0.625 × 0.625
(Luisier et al. 2014)	Femur	HA	QMR	BMD to BV/TV from μCT^j^	E_o_ = 6614	Ultimate Force	Brilliance 64, Phillips	120	100 mA	1.0 × 0.33 × 0.33
						Stance: R^2^ = 0.797 Side: R^2^ = 0.842				
(Enns-Bray et al. 2014)	Femur	NR	NR	ρ_ash_ = ρ_QCT_	E_3_ = 10500ρ_ash_^2.29^ See paper for anisotropic modulus	Axial Stiffness	Discovery CT750HD, GE Healthcare	120	60 mAs	0.625 × 0.625 × 0.625
						Anisotropic: R^2^ = 0.783 Isotropic: R^2^ = 0.792				
						Ultimate Strength				
						Anisotropic: R^2^ = 0.355 Isotropic: R^2^ = 0.350				
(Anez-Bustillos et al. 2013)	Femur	HA	Image Analysis	NR	Experimentally derived	Axial Rigidity	ACQSim, Phillips	120	220 mA	3.0 × 0.9375 × 0.9375
						R^2^ = 0.82				
						Bending Rigidity				
						R^2^ = 0.86				
						Failure Load				
						R^2^ = 0.89				
(Mirzaei et al. 2014)	Femur	K_2_HPO_4_	Mindways	ρ_ash_ = 1.22${\uprho}_{{\mathrm{K}}_2\mathrm{H}\mathrm{P}{\mathrm{O}}_4}$ + 0.0526^b^	E = 33900ρ_ash_^2.20^ for 0 < ρ_ash_ < 0.27 E = 5307ρ_ash_ + 469 for 0.27 < ρ_ash_ < 0.60 E = 10200ρ_ash_^2.01^ for ρ_ash_ > 0.60	Load	Somatom 64, Siemens	140	80 mAs	1.0 × 0.50 × 0.50
						R^2^ = 0.809–0.886 See paper for specifics by method				
(Arachchi et al. 2015)	Femur	HA	NR	NR	NR	NE	Brilliance 64, Phillips & Somatom Plus 4, Siemens	140	206 mAs	2.0 × 0.29 × 0.29
(Kheirollahi and Luo 2015)	Femur	NR	NR	ρ_ash_ = 0.04162 + 0.000854HU	E = 10500ρ_ash_^2.29^	NE	NR	NR	NR	NR
(Carballido-gamio et al. 2015)	Femur	Both	Mindways & Image Analysis	vBMD reported	NR	NE	Lightspeed QX-I, Lightspeed VCT, Lightspeed 16, GE Healthcare & Biograph 16, Siemens	NR	NR	2.0 × 0.742 × 0.742 OR 2.5 × 0.938 × 0.938 OR 1.0 × 0.977 × 0.977
(Kaneko et al. 2015)	Femur	HA	B-MAS200	ρ_ash_ = ρ_HA_	NR	NE	Light Speed Ultra16, GE Healthcare	120	80 mA	NR
(Varghese et al. 2011)	Femur, Tibia, Humerus, Radius	K_2_HPO_4_	Mindways	NR	NR	Strain	Lightspeed 16, GE Healthcare	80	200 mAs	0.625 × 0.625 × 0.625
						R^2^ = 0.61–0.99 See paper for specifics by method				
(Kopperdhal et al. 2014)	Spine & Femur	HA	Image Analysis	BMD related to HU	NR	NE	Somatom Plus 4, Siemens	120	150 mAs	Spine: 1.0 × 1.0 × 1.0 Femur: 1.5 × 1.5 × 1.5
(Kleerekoper et al. 2014)	Spine & Femur	NR	NR	NR	NR	NE	NR	NR	NR	NR
(Keaveny et al. 2014)	Spine & Femur	HA	European Spine Phantom	NR	NR	NE	NR	120	Femur: 170 mAs Spine: 100 mAs	NR
(Zeinali et al. 2010)	Spine	K_2_HPO_4_	Mindways	BMD related to HU	E_z_ = −34.7 + 3230${\uprho}_{{\mathrm{K}}_2\mathrm{H}\mathrm{P}{\mathrm{O}}_4}$ E_z_ = −2980${\uprho}_{{\mathrm{K}}_2\mathrm{H}\mathrm{P}{\mathrm{O}}_4}$ ^1.05^ ${\uprho}_{{\mathrm{K}}_2\mathrm{H}\mathrm{P}{\mathrm{O}}_4}$ = 0.0527 g/cc E_x_ = E_y_ = 0.333E_z_	Strength	Somatom Plus 64, Siemens	140	400 mA	1.0 × 0.25 × 0.25
						Linear elastic–plastic: R^2^ = 0.937 Linear elastic-perfectly plastic: R^2^ = 0.855 Linear elastic: R^2^ = 0.831 Min. sectional: R^2^ = 0.863				
(Tawara et al. 2010)	Spine	HA	B-MAS200	ρ_app_ = 0.0 (HU < −1) ρ_app_ = (0.733HU + 4.51)*10^−3^ (−1 ≤ HU)	E = 0.001 for ρ_ash_ = 0 E = 33900ρ_ash_^2.20^ for 0 < ρ_ash_ < 0.27 E = 5307ρ_ash_ + 469 for 0.27 < ρ_ash_ < 0.60 E = 10200ρ_ash_^2.01^ for ρ_ash_ > 0.60	NE	Hitachi	120	NR	1.0 × 0.39 × 0.39
(Unnikrishnan and Morgan 2011)	Spine	HA	Image Analysis	ρ_HA_ based	E_zz_ = −34.7 + 3.230ρ_HA_ E_xx_ = E_yy_ = 0.333	NE	Light Speed VCT, GE Healthcare	120	240 mA	0.625 × 0.31 × 0.31
(Christiansen et al. 2011)	Spine	HA	Image Analysis	ρ_HA_ based	NR	NE	Light Speed Plus, GE Healthcare	120	100 to 360 mAs	2.5 × 0.68 × 0.68
(Imai 2011)	Spine	HA	NR	ρ_ash_ = ρ_HA_	E_cort_ = 10000	NE	Light Speed QX/i, GE Healthcare	120	360 mA	2.0 × 0.35 × 0.35
(Dall’Ara et al. 2012)	Spine	K_2_HPO_4_	Mindways	BV/TV using the relationships BV/TV = 0 for BMD < −100 BV/TV = 0.0942*BMD-0.0297 for −100 < BMD < 1061 BV/TV = 1061 for BMD >1061	E = 8780	Strength	Brilliance 64, Pillips	120	100 mA	0.45 × 0.39 × 0.39
						hFE: R^2^ = 0.79				
						Failure Load				
						hFE: R^2^ = 0.78				
(Wang et al. 2012)	Spine	HA	Image Analysis	vBMD based	NR	Strength	NR	120	150 mAs	NR
						R^2^ = 0.85				
(Unnikrishnan et al. 2013)	Spine	HA	Image Analysis	BMD related to HU	E_z_ = −34.7 + 3230ρ_HA_ E_z_ = −2980ρ_HA_^1.05^ ρ_HA_ = 0.0527 g/cc E_x_ = E_y_ = 0.333E_z_	NE	Light Speed VCT, GE Healthcare	120	240 mA	0.625 × 0.3125 × 0.3125
(Lu et al. 2014a)	Spine	Both	Mindways & QRM	NR	NR	NE	Sensation 64, Siemens	120	360 mAs	0.60 × 0.32 × 0.32 OR 0.30 × 0.18 × 0.18
(Matsuura et al. 2014)	Spine	K_2_HPO_4_	Mindways	ρ_ash_ = ${\uprho}_{{\mathrm{K}}_2\mathrm{H}\mathrm{P}{\mathrm{O}}_4}$	ρ_ash_ = 0: E = 0.001 ρ_ash_ > 0: E = 1890 ρ_ash_^1.92^	Fracture Load	Somatom Definition, Siemens	120	210 mA	0.40 × 0.30 × 0.30
						R^2^ = 0.78				
						Axial Stiffness				
						R^2^ = 0.39				
(Lu et al. 2014b)	Spine	HA	QMR	BMD related to HU	E_z_ = 2980(ρ_HA_/1000)^1.05^ for ρ_HA_ < 52.7 [mg_HA_/cc] E_z_ = = −34.7 + 3230ρ_HA_ for ρ_HA_ > 52.7 [mg_HA_/cc]	NE	Mx8000, Phillips	90 & 120	100 & 150 mAs	1.3 × 0.30 × 0.30
(Campoli et al. 2014)	Scapula	NR	NR	ρ_app_ = HU + 0.00039	E = 6850ρ_app_^1.49^	NE	Somatom Definition, Siemens	NR	NR	0.6 × 0.6 × 0.6
(Pomwenger et al. 2014)	Scapula	NR	NR	ρ_app_ = 1.1187*10^−3^*HU^k^ assumed ρ_app_ = 0 no bone & ρ_app_ = 1.8 for bone	E = 1049.45ρ_app_^2^ ρ_app_ < 0.35 E = 3000ρ_app_^3^ ρ_app_ > 0.35	NE	NR	NR	NR	NR
(Hermida et al. 2014)	Scapula	K_2_HPO_4_	Mindways	NR	E_cort_ = 20000	NE	NR	NR	NR	NR
(Edwards et al. 2013)	Tibia	HA	QRM	ρ_HA_ = BMD ρ_app_/ρ_HA_ = 0.626	E_3_ = 6570ρ_app_^1.37^ E_min_ = 0.01 E_1_ = 0.574E_3_ E_2_ = 0.577E_3_	Rotation Stiffness	Brightspeed, GE Healthcare	120	200 mA	0.625 × 0.352 × 0.352
						R^2^ = 0.920				
						Ultimate Strength				
						R^2^ = 0.753				
(Nazemi et al. 2015)	Tibia	K_2_HPO_4_	Mindways	ρ_ash_ = 0.55 ρ_app_^g^ ρ_ash_ = 0.597ρ_dry_^g^ ρ_real_ = 1.8 g/cc^l^ ρ_app_ = ρ_real_*BV/TV BMD = 0.904ρ_ash_ - 0.0321^g^ ρ_ash_ = 1.06*BMD + 0.0389^g^		Axial Stiffness	Aquilion 64, Tobisha	120	150 mAs	0.5 × 0.5 × 0.5
					E = 15520ρ_app_^1.93^	R^2^ = 0.75				
					E = 6570ρ_app_^1.37^	R^2^ = 0.65				
					E = 33200ρ_ash_^2.2^	R^2^ = 0.70				
					E = 4778ρ_app_^1.99^	R^2^ = 0.69				
					E = 3311ρ_dry_^1.66^	R^2^ = 0.67				
					E = 3890ρ_dry_^2^	R^2^ = 0.69				
					E = 6310(BV/TV)^2.1^	R^2^ = 0.70				
(McErlain et al. 2011)	Knee	SB3	Gamex	NR	NR	NE	Multistar, Siemens	90	40 mAs	NR
(Synek et al. 2015)	Radius	NR	NR	BMD to BV/TV from μCT	Multiple – Refer to paper	Axial Stiffness	Discovery CT750HD. GE Healthcare	140	260 mA	0.63 × 0.20 × 0.20
						Isotropic-Homogeneous R^2^ = 0.500 Isotropic-Heterogeneous R^2^ = 0.816 Orthotropic-Heterogeneous R^2^ = 0.807				

*HA* Hydroxyapatite, *K*_*2*_ *HPO*_*4*_ Dipotassium Phosphate, *NR* Not Reported, *BMD* Bone Mineral Density, *BV/TV* Bone Volume/Total Volume, *NE* No Experimental; ^a^ (Schileo et al. 2008); ^b^ (Les et al. 1994); ^c^ (Suzuki et al. 1991); ^d^ (Keyak et al. 1997); ^e^ (Keyak et al. 2005); (Faulkner et al. 1993); ^g^ (Keyak et al. 1994); ^h^ (Dall’Ara et al. 2011); ^I^ (Keyak et al. 2005); ^j^ (Pahr and Zysset 2009); ^k^ (Gupta and Dan 2004); ^l^ (Carter and Hayes 1977)

### Densitometric measurements

#### Ash density

Ash density (ρ_ash_) is a measure typically taken on small bone samples, which are used to determine density-modulus relationships mechanically tested as a continuum (Les et al. 1994). It is calculated as the ash mass divided by bulk sample volume. In the method described by Les et al. (1994), physical measurements were taken on cylindrical bone samples to determine the total sample volume. The sample was ashed in a muffle furnace at 800 °C for 24 h, and weighed to determine the ash mass and the ash density is calculated by dividing by the sample volume.

A similar study tested the effect of ashing temperature on sample mass. Öhman et al. (2007) found that ashing their samples at a temperature of 650 °C for 24 h in a muffle furnace, produced little variation in measured ash mass, compared to increased furnace temperature. Temperatures between 600 and 650 °C, produced significant variation in sample mass. Although the original method described by Les et al. (1994) is still most commonly used, more accurate methods of initial volume measurement, such as micro-CT, or laser scanning may be employed.

#### Apparent density

Bone apparent density (ρ_app_) is calculated as the wet mass of a bone tissue sample divided by the total sample volume. To determine wet mass, Galante et al. (1970) first washed samples to remove marrow, immersed samples in distilled water, and degassed under vacuum. Samples were then removed from water, centrifuged for 15 min at 8000 × g and suspended from an analytical balance for submerged mass. Samples were removed and blotted dry and weighed in air for wet mass. Similarly, Keyak et al. (1994) measured bone cubes by first defatting samples in an 8 and 16 h ethyl alcohol bath, followed by an 8 and 16 h ethyl ether bath. Samples dried for 24 h at room temperature and were weighed for dry mass. The cubes were rehydrated under vacuum in water for 24 h, centrifuged at 750 × g for 15 min, and weighed for hydrated mass. Sample apparent density was then calculated with the known cube volume.

#### Tissue density

The tissue density (ρ_tissue_) also uses the wet mass of the sample; however, as the name suggests, tissue density is a measure of the physical bone tissue (excluding pores) (Galante et al. 1970). It is calculated by dividing the wet mass by the volume of bone tissue. To determine the volume of bone tissue Galante et al. (1970) calculated the difference between the wet and submerged mass.

#### Radiological (mineral equivalent) density

Radiological, or mineral equivalent (K_2_HPO_4_ or HA) density (${\uprho}_{{\mathrm{K}}_2\mathrm{H}\mathrm{P}{\mathrm{O}}_4}$, ρ_HA, _or ρ_QCT_) is calculated by sampling the average CT number (HU) value of all voxels within a region of interest of the known calibration phantom sample rods. The radiographic density of the rods can be estimated using the calibration parameters supplied by the phantom manufacturer, and simple linear regression calculations (Les et al. 1994; Schileo et al. 2008). The QCT calibration can be completed on an entire volume, or by individual CT image.

## Results

Of the 55 studies that met the inclusion criteria and were included, 29% reported the use of a K_2_HPO_4_ phantom, 47% an HA phantom, 13% did not report phantom type, 7% reported use of both K_2_HPO_4_ and HA phantoms, and 4% alternate phantom types. The most commonly reported K_2_HPO_4_ phantom was the Mindways Software phantom, and the most commonly reported HA phantom was the Image Analysis phantom. The most common densitometric relationship between ash density and QCT equivalent density was that developed by Les et al. (1994) (13% of studies). Of all studies, 35% report density-modulus relationships based on ash density, and 18% report ash density directly equivalent to QCT density (K_2_HPO_4_ or HA). Of the studies included as part of this review, 24% report density-modulus relationships determined either from micro-CT bone volume/total volume (μCT_BV/TV_), or relate modulus directly to QCT density, through experimental validation (Zeinali et al. 2010; Christiansen et al. 2011; Unnikrishnan and Morgan 2011; Dall’Ara et al. 2012, 2013; Wang et al. 2012; Anez-Bustillos et al. 2013; Kersh et al. 2013; Unnikrishnan et al. 2013; Luisier et al. 2014; Lu et al. 2014b; Carballido-gamio et al. 2015; Synek et al. 2015). Scanner type and/or settings were omitted or only partially reported in 31% of studies. Studies involving the femur were most prevalent (37), followed by the spine (14), scapula (3), tibia (3), radius (1), knee (1), and humerus (1).

Of the studies reporting density-modulus relationships and experimental validation metrics, those with the lowest mean %-difference, lowest relative error, or correlations greater than 90% (R^2^ > 0.90), 5 used relationships based on ash density (Dragomir-Daescu et al. 2011; Trabelsi et al. 2011; Trabelsi and Yosibash 2011; Ruess et al. 2012; Hambli and Allaoui 2013), 3 based on K_2_HPO_4_ calibrated density (Zeinali et al. 2010; Eberle et al. 2013a, b), and 1 based on apparent density (Edwards et al. 2013).

## Discussion

When creating continuum-level finite element models with heterogeneous material distributions, BMD must first be extracted from scan data, and then a density-modulus relationship applied. From the studies reviewed, it is difficult to quantify and isolate the effect of chosen densitometric relationships on experimental versus computational model error because reported results are the combination of two relationships (densitometric and density-modulus). It was therefore the goal of this review to provide the current state of QCT in FE modeling, and provide the most common methods used in the conversion of densitometric measures. When assessing the accuracy of density-modulus relationships developed in previous studies, and comparing experimental to computational results, replication of the density measure and/or accurate conversion between density measures is necessary to reduce inaccuracies and error.

The majority of articles included in this review were studies involving the femur. The hip represents one of the most widely studied joints, and as such, many of the densitometric and density-modulus relationships have been developed using femur specimens. Computational models using femur developed densitometric and density-modulus relationships have shown excellent agreement between experimental models and FEMs (Table 1). This is not the case with other bones/joints that lack relationships specific to each specific anatomical location, or use equations that have been developed using femurs, or femur specimens. Differences between the femur and other bones may reduce the effectiveness of translating these relationships for use in other bones/joints, especially those that exhibit drastically different loading conditions, or mineralization patterns.

A large number of the studies reviewed reported relationships between QCT derived density and ash or apparent density derived in previous studies (Table 1 & Figs. 1 and 2). Ash density was used as equivalent to QCT density in 18% of studies. Schileo et al. (2008) showed that although linearly correlated (R^2^ = 0.997), ash and QCT density are not equivalent. When using densitometric relationships developed in previous studies, it is important to note that the relationships may be a function of the scanner settings and protocol, as well as the anatomical location and pathology of the bone (Faulkner et al. 1993; Kopperdahl et al. 2002; Schileo et al. 2008; Giambini et al. 2015). All these factors may increase the error when then using previously developed bone density-modulus relationships. Giambini et al. (2015) found that reconstruction kernel, as well as tube voltage, had a significant effect on cortical and cancellous QCT derived CT number (HU). This may indicate that even for scans performed on the same scanner, when scanner settings are altered, there may be significant variations in measured CT number, and consequently, material property assignment.

Direct comparison of QCT derived bone density to modulus has the potential to decrease this error, and may improve the accuracy of subject-specific FE models (Kopperdahl et al. 2002). This method minimizes error arising from densitometric conversion, variations in BMD by anatomical location and pathology of bone, and allows for subject-specific material mapping, and density-modulus relationship development. The desired outcome of the FE model should also be noted in choosing a density measure, as BMD corresponds mainly to ultimate strength or modulus, due to its lack of dependence on bone size.

When modeling bone with use of clinical resolution CT, partial volume effects must be taken into account, as well as the averaging of CT lattice vertices in the generated mesh (Taddei et al. 2004). Micro-CT model generation allows for these effects to be minimized, and for the generation of material assignment based on bone volume and mineral density (Dall’Ara et al. 2011; Zysset et al. 2015). However, the clinical availability and feasibility (Poelert et al. 2013), as well as size restrictions and dose of micro-CT limit its use with patient populations, and with larger bones and joints. Giambini et al. (2015) suggest using dual-energy CT to isolate bone from non-bone constituents within the matrix. This method can be implemented on standard clinical CT scanners and provides an interesting framework for future clinical-based FE studies; however, may be less desirable to patient populations due to increased dose requirements.

This review is not to suggest that previously developed models using mechanical testing, and physical density measurements are obsolete or suboptimal, but rather to provide the current state of QCT-based FE modeling, and to suggest that considerations in density mapping be carefully explored before model generation – in particular when using previously developed relationships. In subject-specific modeling, it is important to use empirical density-modulus relationships developed for the same anatomical site in order to increase model accuracy (Zadpoor and Weinans 2015). In using previously developed density-modulus relationships, comparing ash to apparent density, Schileo et al. (2008) determined a conversion factor of ρ_ash_/ρ_app_ = 0.6 be used for both cortical and cancellous bone, to avoid over- or under-estimation of density. This equation was the most commonly used conversion between the two density measures in the studies reviewed, with most studies reporting previously determined density-modulus relationships using ash density. While this conversion provides one value for cortical and cancellous bone, the authors report that this conversion was determined using human femur specimens, and that similar conversions should be developed for alternate anatomical locations, as the structural mineralization of the tissue is dependent on anatomical location and pathology of the bone (Schileo et al. 2008).

The limitations of this study are that an in-depth evaluation of the specific effect of densitometric conversions of FEM outcomes, and specifics of the density-modulus relationships are not discussed. The combination of these two relationships as a requirement for FEM development means they are not mutually exclusive and the effect of one without the other is therefore difficult to assess. We have provided experimental versus FEM validation metrics to allow for the combination of the two relationships to be assessed based on the type of study (Table 1). Specifics regarding the density-modulus relationships are compared and contrasted in the review by Helgason et al. (2008).

The lack of reported scanning parameters used in QCT-based FE studies has been previously stated (Giambini et al. 2015). Many of the studies included in this review lack one or all of phantom type and manufacturer, density and modulus relationships, as well as scanner type and scanner settings (Table 1). Since the combination of these parameters may alter calculated density and subsequent elastic modulus, we suggest that standardized reporting (see Table 1) should be included in future QCT-based FE studies to facilitate comparison with previous findings, and to ensure that methods are repeatable. This has the potential to improve the accuracy of future FE models. When assessing uncertainty in mechanical property assignments in FE models, Laz et al. (2007) provides an excellent framework, which should be incorporated into both experimental and clinical FE models.

## Conclusions

This review assessed the current state of QCT-based FE modeling with use of clinical scanners. It was found that previously developed relationships vary by anatomical location, scanner type and settings. Reporting of all parameters used when referring to previously developed relationships, or in the development of new relationships, may increase the accuracy and repeatability of future FE models. Furthermore, the specific image processing steps in the conversion of raw attenuation data should be included whenever using QCT methods.
